# Study Design and Protocol to Assess Fruit and Vegetable Waste at School Lunches

**DOI:** 10.3390/bs9090101

**Published:** 2019-09-18

**Authors:** Allison Marshall, Gregory Bounds, Krista Patlovich, Christine Markham, Alicia Farhat, Nan Cramer, Amanda Oceguera, Travis Croom, Jamie Carrillo, Shreela Sharma

**Affiliations:** 1 Michael & Susan Dell Center for Healthy Living, The University of Texas Health Science Center (UTHealth) School of Public Health, Austin Regional Campus, Austin, TX 78701, USA; allison.n.marshall@uth.tmc.edu; 2 Department of Epidemiology, Human Genetics and Environmental Sciences, Michael & Susan Dell Center for Healthy Living, The University of Texas Health Science Center at Houston (UTHealth) School of Public Health, Houston, TX 77030, USA; gwbounds@gmail.com (G.B.); krista.patlovich@gmail.com (K.P.); travis.croom@gmail.com (T.C.); 3 Department of Health Promotion and Behavioral Sciences, Center for Health Promotion and Prevention Research, The University of Texas Health Science Center at Houston (UTHealth) School of Public Health, Houston, TX 77030, USA; christine.markham@uth.tmc.edu; 4 Brighter Bites, Houston, TX 77029, USACarrillo.jamie@gmail.com (J.C.); 5 Houston Independent School District Food Service Support Facility, Houston, TX 77028, USA; ncramer@houstonisd.org (N.C.); aoceguer@houstonisd.org (A.O.)

**Keywords:** plate waste, school lunch, dietary intervention

## Abstract

This paper has two main aims: (1) to describe the design, implementation, and testing of a protocol to assess longitudinal changes in F&V plate waste conducted as part of a quasi-experimental study, (2) to provide baseline descriptive data on school demographics and study participants. This paper describes the protocol development and implementation, and presents baseline data of a longitudinal fruit and vegetable (F&V) plate waste study. The protocol was developed to determine the preliminary impact of Brighter Bites, a 16-week school-based nutrition intervention, on F&V wasted and nutrients wasted from school lunches. We measured plate waste using a quasi-experimental design (n = 2 intervention schools receiving Brighter Bites, n=1 comparison school; n = 115 4th and 5th grade children). We measured plate waste for five days at each of four time points over the 2017–2018 academic year (baseline prior to intervention, three additional time points). Data collectors measured lunch F&V waste using digital scales and recorded weights on a data collection app. This study was conducted in three central Texas public elementary schools serving predominantly low-income families (>89% of children on free/reduced lunch program). On average, at baseline, 59.1% of all F&V were wasted and children tried <1 F&V at meals. Foods most wasted were legumes and foods least wasted were par-fried baked potatoes. Final retention rate across the four time points was 75.70%. Measurement inter-rater reliability was 100% (r = 0.99). Our study presents a protocol for detailed, individual-level, longitudinal plate waste assessment in elementary schools.

## 1. Introduction

Across socioeconomic status and gender, fruit and vegetable (F&V) consumption among school-aged children in the United States (U.S.) remains below the recommended guidelines set forth by child health and nutrition experts [1]. In 2007–2010, among children aged 1–18 years, 60% did not meet U.S. Department of Agriculture (USDA) fruit intake recommendations, and 93% did not meet USDA vegetable intake recommendations [2]. Among children, nutrition affects school performance and school absences through illness and disciplinary action due to poor behavior [3]. F&V intake is also associated with reduced risks of heart disease, cardiovascular mortality [4] and some cancers among adults [1,5,6,7]. 

The purpose of this paper is to describe the development and implementation of a longitudinal F&V plate waste study protocol and related lessons learned. In collaboration with two large school districts in Houston and Dallas, Texas, we conducted a pilot quasi-experimental plate waste study in the 2017–2018 school year across three schools in Texas to determine the impact of Brighter Bites, a school-based F&V nutrition intervention, on F&V waste at school lunches. Results of a Brighter Bites program evaluation demonstrated significant improvements in the intake of F&V among participating children and parents, and improvements in the home nutrition environment [8]. This protocol is important because it provides a detailed, longitudinal, individual-level estimate of the amount and type of F&V wasted on the child’s lunch. Many plate waste studies use bulk measurement by combining all food waste and taking one weight for an entire group [9,10,11]. Direct measurement of plate waste data collection is more objective than self-report dietary intake measures [12]. It is superior to visual observations, which are less sensitive to detecting change in plate waste [13].

In the United States, the National School Lunch Program (NSLP) was established to provide nutritious foods and facilitate institution of lunch programs in schools across America including through purchase assistance and reimbursement [14] The original programs were intended to be non-profit programs, serve free- or reduced-price lunches to children in need and to meet 1/3 to ½ the minimum nutritional requirements of a 10–12 year-old child [14]. 

The NSLP currently operates in 94% of all public and non-profit private schools in America and is in place in roughly 100,000 public, nonprofit private schools, and residential care institutions, providing free or low-cost lunches to more than 30.4 million students [15,16]. Of students participating in the NSLP, an estimated 68.2% participate in the free/reduced-price lunch program [15]. Because many students rely heavily on school meal provision for 1/3 to ½ of their daily caloric needs or more, the quality of foods available in schools is influential in the dietary quality and overall health of children [15]. 

In response to the Healthy Hunger-Free Kids Act of 2010 and the Dietary Guidelines for Americans, schools serving the NSLP for grades K-5, children ages 5–10, are required to offer ½ cup of fruit and ¾ cup of vegetable daily, as well as meeting vegetable sub-group requirements in order for that meal to be reimbursable at the school level [17,18]. All students in a school participating in NSLP are eligible to participate in the NSLP through by purchasing a school lunch. Children from households with incomes at or below 130 percent of the Federal Poverty Level (FPL) qualify for free lunches, and children from households with incomes 130–185 percent of the FPL are eligible for reduced-price lunches [16]. Lunch purchase takes places through a computerized system using a student ID number or scanned card linked to an online account on which funds may be uploaded electronically. Students who are eligible for free or reduced-price lunch are either not charged at all, or are charged less than students who pay full price. Thus, all students receiving a school meal go through the same process in person, regardless of price paid (free, reduced-price, or full price). 

While many children rely on the NSLP for nutrient and caloric intake, previous plate-waste studies have demonstrated that children may not consume sufficient F&V in their school lunches, due either to not choosing them in the lunch line or leaving them untouched on their tray resulting in insufficient quantities of fiber, and vitamins and minerals being consumed [19,20]. Plate waste of school lunches results in nutrients wasted [19] and unnecessary costs to the NSLP including costs of disposal, over-purchasing, and inefficient resource allocation for the production and transportation of food items wasted [9,10]. Thus, strategies to reduce F&V waste at school lunches are needed. 

A systematic review of food waste in the NSLP from 1978–2015 indicates that there is great diversity in methods and measures for food waste and that consistent food waste measures and methods are needed for comparison across studies [21]. The protocol presented in this paper allows for assessment of individual student F&V waste and consumption. This paper has two main aims: (1) to describe the design, implementation, and testing of a protocol to assess F&V plate waste, (2) to provide descriptive data on school demographics and study participants, as well as differences according to school and school district at baseline.

## 2. Materials and Methods

### 2.1. Plate Waste Study Design

We assessed the preliminary impact of the Brighter Bites school-based intervention on amount, type and variety of F&V selected and wasted by participating students for school lunches over the school year through weighing of individual student F&V waste, direct observation, technology in the form of a data collection app, and qualitative methods inclusive of field notes. Outcomes of this plate waste study are presented elsewhere [22]. This paper will present the development and testing of a protocol to assess plate waste and baseline data from the outcome evaluation study. A pre-post quasi-experimental design was employed to determine the impact of the Brighter Bites intervention on reducing plate waste at school lunches. We conducted our plate-waste study in the 2017–2018 school year as part of a larger outcomes evaluation study to assess the preliminary impact of Brighter Bites on plate waste of F&V in school lunches among fourth and fifth grade students across three large, urban public elementary schools in Houston and Dallas. Two of these schools were receiving Brighter Bites in the 2017–2018 school year, and one was a comparison school that did not receive Brighter Bites (usual care). We hypothesized that from baseline to mid-point (end of 8 weeks), and post-intervention (end of 16 weeks), children receiving Brighter Bites would demonstrate increased selection and decreased waste of F&V in school lunches as compared to those in the comparison school not receiving Brighter Bites. Overall, the results of our pilot plate waste study demonstrated significant decrease from baseline to end of intervention in the amount of F&V and related nutrients wasted among children participating in Brighter Bites, as compared to children not participating in the program [22].

Data was collected at four time points per child during the 2017–2018 school year. Trained data collectors measured participating students’ school cafeteria lunch at two schools participating and one school not participating in the Brighter Bites program. Measurements took place every day (Monday-Friday) of the week prior to the first week of Brighter Bites programming (baseline), mid-point of the program (end of 8 weeks of Brighter Bites distribution in the fall), beginning of the spring Brighter Bites programming, and at the end of the 16-weeks of the Brighter Bites program (end of spring). While all 4th and 5th grade children in the participating intervention schools received the program, only those consenting to the study were measured. This study was approved by the University of Texas Health Science Center, Committee for Protection of Human Subjects.

### 2.2. Brighter Bites Program Overview

Brighter Bites is an evidence-based ongoing 16-week school-based nutrition program consisting of three main components: (1) Weekly distributions of 50 servings of fresh, donated F&V procured from the local food bank, and sent home with parents; (2) Nutrition education which includes evidence-based Coordinated Approach to Child Health (CATCH) program in schools, and parent education via bilingual nutrition handbooks and recipe cards [8,23], and (3) Recipe demonstrations of produce given in the bags for parents and children and a health-focused message for parents at produce pick-up time. The program is implemented for 8 weeks in the fall and 8 weeks in the spring semester. 

### 2.3. Selection, Recruitment, and Participants

#### 2.3.1. Inclusion and Exclusion Criteria—Schools

Using demographic information available from district reports including Title I status of schools, racial/ethnic composition, and percentage of the student population eligible for the free/reduced-priced lunch program, a convenience sample of two school districts in Houston (District A) and Dallas (District B), Texas were recruited to participate in the study. Two of these schools were receiving Brighter Bites programming for the first time in the 2017–2018 school year, one from each district, and the comparison school was not receiving, and had never prior participated in Brighter Bites.

Inclusion criteria—Students: (a) only intervention schools: enrolled in Brighter Bites in the 2017–2018 school year, (b) participating in the NSLP at the school, (c) enrolled in 4th or 5th grade in the 2017–2018 school year.

Exclusion criteria—Students: Prior participation in Brighter Bites. 

#### 2.3.2. Recruitment and Retention

Recruitment: A total of 115 students were recruited and consented at the three schools (intervention school 1: N = 44, intervention school 2: N = 32, Comparison School: N = 39). All forms were available in Spanish and English. Written informed consent was obtained from parents of the participating children. Consent forms were sent home with the child along with the Brighter Bites opt-in form at the beginning of the school year at intervention schools 1 and 2. Child assent forms were sent home and returned in the same packet with consent forms. Once consent and assent were obtained, unique study identifier numbers were assigned by project staff to each child who consented to participate in the study. In the comparison school, consent and assent forms were distributed at the beginning of the school year along with initial start-of-school paperwork. 

Retention: All children were measured for five days (the entire school week) for each time point. Children who brought a home lunch were recorded in field notes but no food items were weighed. Children who went through the cafeteria line and received a school lunch were measured, regardless of type of payment (free/reduced-lunch or full-price); data collectors and other students were unaware of free/reduced-price lunch eligibility of students. Children who had less than three days of school lunch measurements at baseline were excluded from the subsequent measurements (N = 14, 20.9%). In the control school, six days of data collection were conducted due to an unscheduled citywide holiday which did not affect the two intervention schools. We chose to measure the 6th day, only under specific circumstances if the school was closed on one day in the week of measurement, or if a >25% of the participating students were absent due to illness (or other reasons) in any of the schools. We recorded students’ presence or absence in school. At one school with high home-lunch rates, we sent home reminders to participating families that the goal of our study was to evaluate school lunches and to please purchase school lunches.

### 2.4. Protocol

#### Simulation and Training—Development and Pretesting of Protocol

The primary outcome of the study is change in the selection and amount of F&V wasted at school lunch. We used two types of data collection measures for the plate-waste study: (a) Type of F&V selected and weighing the F&V waste on the school lunch tray, and (b) Field notes—these were qualitative data to record the child behaviors and the school environment as observed by the data collector during the school lunch. Detailed description of data collection process and measures are described below. 

### 2.5. Study Protocol Development and Training

We conducted a two-step process to develop our plate-waste protocol:

Step 1. We first simulated a typical school lunch tray and lunch eating experience with five elementary school age children on our university campus. Study PI (Sharma), a registered dietitian with over 10 years’ experience in measurement of dietary intake among children, developed the protocol and training procedures. Fifteen data collectors attended the training. All data collectors observed the children’s lunch behaviors and measured the amount of F&V on the trays before and after the children ate. At the end of training, inter-rater reliability of the data collectors participating in the trainings was measured against study PI measures (reliability >0.95). This helped inform development of the protocol, primarily with regards to the weighing of the amount of F&V, identification strategies for lunch trays and participants, as well as behavioral aspects to note during field observation (i.e., children mixing foods). An important aspect of this was establishing a strategy to identify child participants and their respective trays using numbered stickers and badges after observing potential for students to switch trays or ID numbers. These methods reduced the potential for children to switch or lose numbers for both the participant and the tray. To reduce potential loss of badges, students were given their ID badges immediately prior to lunch. 

Following this, a total of 15 data collectors were trained by study investigators. Initial training was conducted over an eight-hour day in person using project materials including digital scales, Styrofoam bowls, field notes forms, lunch trays, real food items, and children of faculty and staff. Training began with instruction on use of the digital scales, including cleaning, calibration, and demonstrations of use with example food items. Then food items were distributed to children in lunch trays with each data collector assigned to observe the eating patterns of 5 children. The children had been instructed to eat normally, including sharing, trading, or mixing of foods. Data collectors observed the eating behaviors and recorded notes using a field notes form, which were then reviewed as a group to assess for consistency and clarity, and to identify challenges in observation and strategies to overcome them. 

Step 2. Data collectors conducted an observation onsite at participating schools to observe school lunch logistics with the school nutrition services staff. School lunchroom logistics vary by school and by day. Observation of lunch and traffic flow was essential for preparation and planning for observation during data collection. This informed the finalization of field observation strategies in the lunchroom. 

These aforementioned strategies informed the finalization of the F&V plate waste weighing protocol, field observation notes, and data collection app. Prior to full implementation of the protocol, observation and practice sessions were conducted in each school to finalize logistics. The data collection team consisted of a lead data collector/research coordinator and 4–6 graduate research assistants for each day of data collection. A lead data collector established stations for the study team based on the number of lunch lines and the flow of students, as well as oversaw all measurements for consistent data collection across all time points. 

### 2.6. Data Collection Measures

Digital scales for F&V plate waste: Objective measures of plate waste from students’ trays were achieved using Schuler Scientific SSP-1502 digital scales (Schuler Scientific, Englewood, CO, USA). Scales were cleaned and calibrated prior to each data collection. Prior to lunch, cafeteria staff provided data collectors with samples of all available choices of F&V options for that day. Data collectors generated standardized weights by weighing three sample portions served by cafeteria staff and averaging the weights for each F&V. District food services provided recipes for all samples measured to allow for nutrient analysis. At the school level we recorded the number of F&V items offered each day of data collection to calculate the average number of F&V items offered on a daily basis, and to compute the proportion of F&V selected versus available. After lunch, data collectors weighed the total amount of each F&V food item wasted on students’ plates to calculate the amount of the item consumed by the student. Data was measured to the nearest 0.01 g. Trained data collectors removed each F&V item from the tray using a spatula to scrape out the container in which it was initially distributed, and transferred the item into a new Styrofoam bowl. 

**Figure 1:**
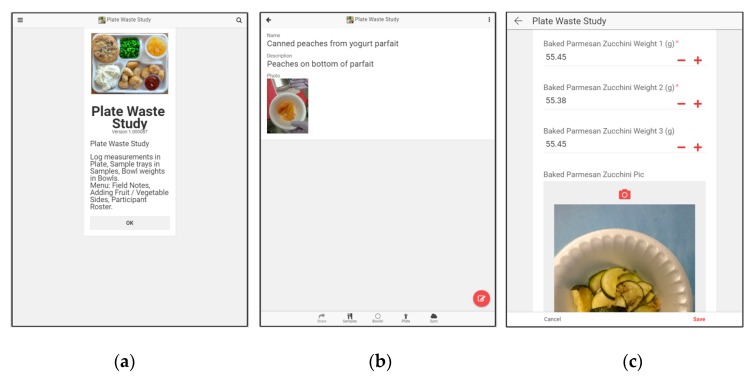
App Screenshots. These screenshots depict the user experience within the app. Initial welcome screen with brief reminder of menus (**a**), a sample fruit and vegetable (F&V) item entry including a name, description, and photograph of the sample (**b**), as well as a lunch tray measurement entry (**c**) which includes a record of the data collector entering item weights and a photo. Additionally, each tray record includes all F&V items selected by the student, an item status (entirely consumed or not eaten) if not weighed, as well as relevant notes.

Data Collection App: We recorded all F&V plate waste data electronically on a data collection app (Google App Scripts) (Google, Mountain View, CA, USA) developed for the project to reduce potential for error and reduce time necessary for recording data on paper and subsequent data entry and cleaning. We developed a mobile application using the Appsheet product to log plate waste data directly into our database (Figure 1a,c). This real-time data entry supported accuracy, real-time monitoring of possible issues and project status, data validation fields to prevent typing errors and require conditional responses, and in-app notifications for ambiguous or inconsistent data. The app included a function to allow data collectors to take photographs and notes of specific items for later reference (Figure 1b). The app also allowed data collectors to record student status (absent, did not purchase a school lunch, or did not purchase any F&V item) as well as item status (not eaten, or entirely consumed) which captured whether or not a student had tried an item at all. 

After the initial wave of data collection, a team member entered F&V recipes provided by school nutrition services into nutritional software (Nutritional Data System for Research [NDSR]) so nutritional values could be analyzed. These data were continuously updated and merged into the dataset as the project continued using Stata 14.2 (StataCorp, College Station, TX, USA).

Field notes: Field notes consisted of qualitative data recorded by data collectors using direct observation during the school lunch. Trained data collectors recorded the following in their field notes: all items initially selected by the students, trading of F&V items, whether or not a student tried or finished their F&V items, if there was any additional food brought from home, if any a la carte food items were purchased, overall eating behavior, as well as the overall school lunch environment. Moreover, behaviors such as: if students got in trouble, were talking with other students, if they were throwing food or otherwise playing with their food, if they were given food by other students (participants, or non-participants), if they were distracted by other activities such as reading, were also recorded. Data collectors observed school food environment aspects such as televisions in the cafeteria, disciplinary action, parents or teachers eating with students, or other school-wide events. 

### 2.7. Data Collection Protocol

On the day of the measurement, before lunch, trained data collectors delivered badges with unique identifier numbers (UIDs) to participating students prior to lunch. Data collectors went to each individual classroom and pinned ID badges on all participating students. Teachers were apprised of the ID badge process and provided with a list of participating students prior to the day of data collection so as to minimize class disruption, and to provide them with an opportunity to talk to their students about the process. Data collectors instructed students to wear the badges during lunch time and reminded students not to throw away their tray after eating. All participants were assigned to a data collector prior to the beginning of the lunch period who recorded all notes on a hard copy field notes form which included the unique ID number for each participant, the date, the name of the data collector, and the name of the school. Data collectors were stationed at the exit to the lunch line to place ID stickers on participants’ trays and the remaining data collectors were stationed around the lunchroom to ensure that all participants could be observed at all times. 

Prior to lunch, cafeteria staff provided data collectors with samples of all available choices of F&V options for that day. Number and type of F&V options available and their weights were recorded per protocol in the data collection app.

During lunch, trained data collectors recorded the child behaviors and school lunch environment in their field notes. Each data collector was responsible to observe up to ten children at one time during each school lunch. Participating students were reminded again to leave their trays on the table following completion of their meal. Data collectors were assigned students to observe by classroom and ID numbers prior to each lunch wave, as students typically ate lunch grouped together by class. In the event that students were not seated together due to disciplinary actions or school-wide events, lead data collectors coordinated in the field so that each student was observed by at least one data collector. Data collectors were specifically instructed to minimize contact with students and research staff did not implement any disciplinary measures. Data collectors were instructed to tell students that they were measuring some things at lunch and to instruct the students to return to their seats if they got up to engage with the research staff. 

After lunch, data collectors collected the trays from the student tables and brought all trays to a separate weighing station where they weighed the type and total amount (in grams) of each F&V food item left on students’ plates per protocol. They also recorded whether or not an item had been tried at all, not eaten, or entirely consumed. All data were recorded on the electronic data collection app. 

#### Inter-rater Reliability Testing

Field notes and F&V item weights were tested for inter-rater reliability (IRR) against a lead data collector during Waves 2 and 3. A total of seven unique data collectors were included in IRR over a total of three days in the field. Five trays were included from an intervention school and five trays were included from the comparison school for both the weight and field notes IRR testing. There were three lead data collectors across the length of the project, all of whom had prior experience in data collection in schools and were trained and validated by senior author and PI Sharma who has extensive experience in collection of dietary data among children. There was partial overlap between inter-rater reliability testing of plate waste and field notes observations, but the same data collector was not measured two days in a row. Student trays were selected at random for inclusion in the IRR testing. 

Agreement between data collectors was scored using percentage agreement. Plate waste measurement was analyzed for inter-rater reliability using 19 F&V items on ten students’ trays across three days in two schools (one intervention school and the comparison school). Field notes were tested for accuracy on 20 F&V-related observations from ten students’ trays against a lead data collector across three days at two schools (one intervention school and the comparison school). Each F&V-related observation was for a single F&V item, with two F&V items per lunch tray. 

### 2.8. Data Analysis 

For this paper, we analyzed the enrollment data, and calculated retention rates as proportions of students for whom we had collected baseline measurements each week at each school. We analyzed baseline data using descriptive statistics such as means, standard deviations and frequencies, including number of daily F&V choices offered per school, and average number of F&V items tried per child, and daily means of F&V food waste, percent of items wasted, sample weights of items, number of items chosen per student, and number of items tried per student. Analysis was performed using Stata 14.1 (Statacorp, College Station, TX, USA). Choice selection and ambiguous data were confirmed with qualitative data documented in the field notes. 

## 3. Results

### 3.1. Results

We targeted demographically similar schools with a high proportion of free and reduced lunch eligibility (90%) to ensure elevated school lunch purchase. All three schools had mostly Hispanic students enrolled with total populations of over 700 students (Table 1). We recruited from 4th and 5th graders at intervention school 1 and had 19.1% (N = 44) of the eligible student’s consent. We recruited 5th graders at intervention school 2 and control school which had 17.5% (N = 32) and 39% (N = 39) of eligible students consent, respectively. After the baseline measurement week (Wave 1), a total of 24 students were removed who did not meet the baseline requirement of at least 3 school lunch measurements at baseline. One student withdrew from the study, and four others left their respective school. The retention rates at Waves 2, 3, and 4 were 84.1% for intervention school 1 and 81.3% at intervention school 2. Retention rates were lower in the control school at 64.1% at Wave 2 and 61.5% at Waves 3 and 4 but not significantly (*p* = 0.96) (Table 2). Overall retention rate at the end of the second 8-week session was 75.70%. 

Table 3 shows the results of the baseline plate waste data collection across the three participating schools. The data are stratified by each school, and the two school districts. For the control school, there were six days of measurement due to a district-wide change in school calendar which did not affect either of the intervention schools. Overall, across the three schools, results show that there were 4.48 F&V choices available and students tried an average of 0.91 items. On average, 59.1% of all F&V were wasted. The most wasted foods were legumes and the least wasted foods were par-fried baked potatoes (data not shown in tables). Table 4 shows all F&V items served and analyzed at baseline. 

### 3.2. Tables

#### Inter-Rater Reliability

The weighing aspects of the data collection protocol showed high Inter-rater reliability. Of the 19 F&V items, all items were recorded correctly by all raters (100% agreement), and the average weight difference was 0.012 g between raters (r = 0.99). The field notes observation component of the data collection protocol showed high Inter-rater reliability. Each of the 20 F&V related observations was of a single F&V item from a lunch tray, with each tray including two F&V items. Of the 20 F&V-related observations, there was 95% (19) agreement. The discrepancy in the field notes observations was a misidentified F&V item. 

## 4. Discussion

Overall, our study provides a detailed description of the study protocol and application for longitudinal measurement of school plate waste to assess the impact of Brighter Bites nutrition intervention on reducing F&V waste at school lunches. Baseline descriptive data are presented to demonstrate this application of the protocol for tracking individual-level, and provide a description of the lunch experience of the study sample at baseline. Prior plate waste studies have provided important data on food waste at school lunches. For example, among middle-school students in Boston, it was found that students consumed less than the recommended amounts of nutrients, and discarded 19% of their entrees, 47% of their fruit, 25% of their milk, and 73% of their vegetables [19]. Latest available USDA data on plate waste found that students wasted 12% of foods served in the NSLP [24]. However, there is a lack of detailed study and measurement protocols for plate-waste studies, especially those that are longitudinal. In addition to F&V waste and related nutrient loss, our protocol provides distinction between the number and type of F&V choices available, whether a participant selected an item, whether they tried it, and whether they finished it. This distinction is important because child exposure to F&V is linked to consumption [25] and increased familiarity and preference of F&V, which in turn is linked to increased consumption of F&V [26,27]. On this point, our study aims to capture if students are indeed showing increased preference through selection and then trying of F&V items. Over time, this protocol can be used to track if students’ selection, trying, and finishing of F&V items increases. Baseline data from our study shows that more than half of the F&V that the children chose were wasted. Furthermore, we are able to obtain detailed information on the type of F&V most and least wasted. These data could be critical to inform foodservice planning and operations at the schools. Next steps are to conduct the outcome analysis to determine the preliminary impact of Brighter Bites on reducing F&V waste at school lunches. Given the plethora of school-based nutrition interventions [28,29] and the validity and reliability limitations of self-report data [29], plate-waste protocols such as ours should be considered to provide consistent and accurate estimates of the impact of these interventions on dietary behaviors of children. 

Our collaboration with local school districts was integral to the successful development and implementation of our plate waste protocol. Through this collaboration we were able to partner with school administration and staff, gather information about recipes, menu planning, and cafeteria logistics. Future studies should be predicated upon buy-in from school districts and administration. Emphasis should be placed on the mutually beneficial nature of plate waste studies in contributing to increased understanding of nutrient loss and intake, student purchasing and consumption habits, and potential to optimize school food service resource allocation. 

Many prior plate waste studies either use bulk measurement, combining all food items for single individuals or for groups of people (such as a lunch wave or grade) [11,20,21]. Bulk measurement fails to measure consumption of individual items or consumption at the individual level. Our pilot study protocol includes measures specific to each individual item, including nutrient loss and is focused specifically on F&V, both of which distinguish it from other plate waste studies. Also, our inter-observer reliability for the plate waste weighing and field notes was high (>95%). Furthermore, the qualitative data from the field notes and interviews helps contextualize the findings. Pre- and post- measurements of plate waste at the individual child level are more sensitive to change than visual observations [13] and are the most precise method of measuring consumption [30]. This study combines visual assessment through field notes and direct observation during lunch, as well as objective measurements of consumption through weighing samples and plate waste. Thus, our method of direct observation could be used to enhance understanding of student choice and consumption behavior of F&V in school cafeterias. The individual-level, item-specific data allows researchers and food services to identify which items are being chosen by students, which items are being tried by students, which items are being entirely consumed by students, and which items are most often wasted by students. We expected greater participation in the NSLP due to high eligibility rates for participation in the free/reduced-price lunch program. Although all three schools were similar in terms of demographics, we found high variability in school lunch participation habits across schools. Future longitudinal studies should incorporate strategies to specifically assess school lunch participation habits among the target population to optimize sample selection. Additionally, efforts can be made to remind students not to bring lunches from home by sending home informational flyers the week prior or informing teachers to promote this strategy on the days preceding the study. 

### 4.1. App-Based Data Collection

To the best of our knowledge, only one previous study utilized a mobile application to measure plate waste in the field. In that study, participants took photographs before and after food consumption outside of a school lunch setting and researchers used a reference point and visual estimations to measure waste [31]. Although visual estimates can be accurate and require less work for data collectors, images must still be reviewed and food nutrients estimated based on food descriptions. Our use of a mobile application focused on improving efficiency of the more common practice of measuring school plate waste where recipes and serving sizes are standardized. The use of an app allows for collecting real-time, detailed, accurate measurements of plate waste at the individual level, and reduces back end data entry error and time. Also, using a data collection app can be scalable for larger studies and can be used for longitudinal studies to track F&V waste over time at the individual level.

### 4.2. Challenges and Considerations for Future Studies

Our study had several challenges and limitations that need to be considered for future studies. Our study sample size was small (n = 3 schools; 115 children), and we relied on a convenience sample. Schools were not randomized, nor were participants randomly assigned to intervention or comparison. There may be differences between those who chose to participate and those who did not choose to participate. Participants were aware they were being observed by study staff on a recurring basis. The presence of study staff could impact typical lunchroom behavior. However, data collectors were strictly informed to not talk or interact with the children during lunch. Social desirability could play a role and that is a limitation of the study. However, due to limitations of resources we could not conduct a fully blinded study which may be considered in the future. While our recruitment rates were high, retention rates in our control school were lower than expected. Retention rate was defined as 3 or more observations per child per time point. So, if the child did not eat a school lunch (i.e., brought lunch from home) which was the majority of the children that we were unable to measure (20.9%), was absent, or inadvertently threw away their lunch tray, then they were not measured for that day. In our study, we tried multiple strategies to facilitate retention including sending multiple reminders home to parents regarding the purpose of the study. Children in the free/reduced-price lunch program received all school meals for free or for reduced-pricing at the time of purchase, with no need for individual reimbursement; this allows children from low-income families access to critical nutrition. However, no financial incentives were offered to the children or families for participation. In our subsequent study, we will consider strategies such as financial incentives (i.e., gift cards), and offering an alternative intervention for the control school children and families to facilitate retention. Another consideration is the personnel expense related to our data collection methods. However, self-report data on dietary intake among children is riddled with issues including social desirability bias and validity issues. Using direct observation and objective weighing of F&V minimizes these errors to provide valid, accurate measurements. Systematic two-step onsite (at schools) and offsite training of our data collection staff followed by inter-observer reliability testing on 10% of the study sample was critical to protocol success. Our protocol inter-observer reliability for the plate waste weighing and field notes was high (>95%), which required substantial resources and coordination during field data collection. The lack of IRR testing for all data collectors is a limitation of these findings. Future studies may consider planning for more thorough IRR testing during each wave as possible. Also, using technology to facilitate data collection on field minimized backend data entry errors and costs. Finally, our observations were limited to school plate waste and may not reflect a child’s school-day food consumption in home lunches, snacks, or in settings outside of school. Even within this small sample size we saw much variation in the number and types of F&V items offered between schools. Future studies may consider efforts to align menu offerings such that different schools have more comparable types of F&V items and number of F&V items during data collection. However, the purpose of the study was to determine the preliminary impact of a school-based nutrition intervention on reducing F&V waste at school lunches, which was successfully achieved. 

## 5. Conclusions and Implications

In conclusion, this study presents the protocol to conduct a detailed, individual-level, longitudinal child plate waste assessment in elementary schools serving predominantly low-income children and their families. Strong collaboration with the foodservice team at both school districts, an onsite and offsite training protocol for data collectors, using technology to facilitate data collection, and the use of mixed-methods qualitative and quantitative assessments can help guide future studies in this area. 

## Figures and Tables

**Table 1:** School Demographics and Study Participants.

	Intervention School 1	Intervention School 2	Control School
Free/Reduced Lunch *	90.0%	90.6%	89.5%
Race/Ethnicity			
Hispanic	70.1%	84.4%	81.5%
Black or African American	20.0%	7.4%	12.6%
White	8.9%	2.2%	1.2%
Other	1.0%	6.1%	4.7%
Total Enrollment	729	775	724
4th	115		
5th	115	183	100
Enrolled in Study	44 (19.1%)	32 (17.5%)	39 (39.0%)

Note: * Based on 2016–17 District Data from Houstonisd.org, Dallasisd.org.

**Table 2:** Participant Retention Rate.

	Baseline	End of First 8-Week Session	Beginning of Second 8-Week Session	End of Second 8-Week Session
Intervention school 1 ^1^	44	84.10%	84.10%	84.10%
Intervention school 2 ^2^	32	81.30%	81.30%	81.30%
Control school ^3^	39	64.10%	61.50%	61.50%
Overall	115	76.50%	75.70%	75.70%

Note: 1 One student withdrew from the study during Wave 2, six students did not meet minimum measurement requirements (at least 3 measurements in Wave 1). 2 Two students left school after Wave 1, four students did not meet minimum measurement requirements (at least 3 measurements in Wave 1). 3 One student left school after Wave 2, fourteen students did not meet minimum measurement requirements (at least 3 measurements in Wave 1).

**Table 3:** Food Waste Descriptives stratified by school and school district at baseline, 2017–2018.

	Day 1		Day 2		Day 3		Day 4		Day 5		Day 6		Wave 1 Average *	
	Mean	SD	Mean	SD	Mean	SD	Mean	SD	Mean	SD	Mean	SD	Mean	SD
Overall (n = 3 schools)														
Number of F&V Choices available	4.37		4.34		3.87		6.04		5.24		3.00		4.48	
Amount F&V Wasted Average (g)	39.25	40.24	38.0	35.65	45.14	32.52	76.87	50.01	75.99	59.04	58.70	37.25	55.95	48.34
F&V Sample Average (g)	88.20	26.24	84.17	24.63	76.07	18.29	90.90	31.16	115.88	61.33	82.1	26.50	92.54	39.39
F&V Wasted Average/Sample Average (%)	48.04	40.80	48.20	42.47	58.38	40.32	75.04	33.48	64.54	36.64	63.72	38.50	59.1	39.91
Average Number of F&V Items chosen	1.71	0.60	1.11	0.72	1.07	0.43	1.12	0.57	0.91	0.42	1.13	0.35	1.32	0.64
Number of F&V Items Tried per Student	1.44		0.91		0.84		0.60		0.75		0.93		0.91	
Schools														
Intervention school 1														
Number of F&V Choices available	5.00		6.00		5.00		8.00		6.00				6.00	
Amount F&V Wasted Average (g)	61.24	48.99	48.76	36.48	33.68	30.46	62.01	48.52	98.51	66.78			65.30	55.08
F&V Sample Average (g)	101.86	28.15	92.36	28.43	79.85	19.25	83.70	30.96	145.04	67.86			101.83	46.39
F&V Wasted Average/Sample Average (%)	59.32	36.83	54.04	38.63	41.10	34.32	75.09	32.89	63.90	36.02			60.62	36.97
Average Number of F&V Items chosen	1.56	0.80	0.69	0.69	1.03	0.51	0.87	0.57	0.87	0.34			1.13	0.65
Number of F&V Items Tried per Student	1.30		0.59		1.00		0.46		0.72				0.81	
Intervention school 2														
Number of F&V Choices available	5.00		4.00		3.00		5.00		5.00				4.40	
Amount F&V Wasted Average (g)	25.96	26.78	38.6	33.41	49.47	36.19	104.37	47.75	61.64	34.79			52.42	44.47
F&V Sample Average (g)	72.25	6.96	70.16	11.730	77.77	10.42	102.04	36.08	103.2	37.73			86.05	27.65
F&V Wasted Average/Sample Average (%)	41.45	43.42	54.03	44.908	62.73	44.44	74.23	28.72	74.86	38.61			58.84	42.61
Average Number of F&V Items chosen	1.88	0.45	1.26	0.53	1.11	0.42	1.25	0.44	0.92	0.41			1.46	0.59
Number of F&V Items Tried per Student	1.50		0.88		0.88		0.88		0.67				0.96	
Control school 3														
Number of F&V Choices available	3.00		3.00		3.00		3.00		4.00		3.00		3.17	
Amount F&V Wasted Average (g)	41.59	40.48	36.85	42.54	85.00	27.53	75.72	39.51	60.02	36.49	63.72	38.50	45.97	38.30
F&V Sample Average (g)	73.79	21.31	78.25	19.03	62.47	22.71	103.52	21.56	68.14	18.55	82.10	26.50	76.63	22.41
F&V Wasted Average/Sample Average (%)	41.59	40.48	36.85	42.54	85.00	27.53	75.72	39.51	60.02	36.49	63.72	38.50	57.21	41.56
Average Number of F&V Items chosen	1.73	0.45	1.50	0.66	1.10	0.30	1.41	0.50	0.96	0.54	1.13	0.35	1.47	0.59
Number of F&V Items Tried per Student	1.54		1.39		0.52		0.55		0.87		0.93		0.97	
Districts														
District A (intervention school 1)														
Number of F&V Choices available	5.00		6.00		5.00		8.00		6.00				6.00	
Amount F&V Wasted Average (g)	61.24	48.99	48.76	36.48	33.68	30.46	62.01	48.52	98.51	66.78			65.30	55.08
F&V Sample Average (g)	101.86	28.15	92.36	28.43	79.85	19.25	83.70	30.96	145.04	67.86			101.83	46.39
F&V Wasted Average/Sample Average (%)	59.32	36.83	54.04	38.63	41.10	34.32	75.09	32.89	63.90	36.02			60.62	36.97
Average Number of F&V Items chosen	1.56	0.80	0.69	0.69	1.03	0.51	0.87	0.57	0.87	0.34			1.13	0.65
Number of F&V Items Tried per Student	1.30		0.59		1.00		0.46		0.72				0.81	
District B (intervention school 2 and control school)														
Number of F&V Choices available	4.00		3.49		3.00		3.98		4.35		3.00		3.64	
Amount F&V Wasted Average (g)	26.55	27.30	32.62	34.23	53.98	31.51	92.45	47.06	49.83	33.37	58.70	37.25	48.95	41.33
F&V Sample Average (g)	72.83	12.57	73.63	14.43	71.21	17.12	102.59	29.62	84.07	32.81	82.1	26.50	81.55	25.44
F&V Wasted Average/Sample Average (%)	41.52	41.74	45.2	44.28	71.71	39.79	74.99	34.35	65.29	37.62	63.72	38.50	57.96	42.00
Average Number of F&V Items chosen	1.80	0.45	1.37	0.60	1.10	0.37	1.33	0.47	0.94	0.47	1.13	0.35	1.46	0.59
Number of F&V Items Tried per Student	1.52		1.14		0.70		0.72		0.77		0.93		0.96	

* Wave 1 average includes six days for the control school only. There were six days of measurement due to a district-wide change in school calendar which did not affect either of the intervention schools. We chose to measure the 6th day, only under specific circumstances if the school was closed on one day in the week of measurement, or if a >25% of the participating students were absent due to illness (or other reasons) in any of the schools.

**Table 4:** Fruit and Vegetable Items Served at Baseline.

School	Day	Description of F&V item
Int 1	1	Apple sauce with fresh cut apple chunks on top
Int 1	1	Fresh, whole Asian pear
Int 1	1	Fresh banana; peel not included in weighing
Int 1	1	Oven baked white potato fries seasoned with garlic
Int 1	1	Baked beans; strained before weighing
Int 1	2	Canned diced mango
Int 1	2	Corn seasoned with cilantro, cheese, chili powder
Int 1	2	Peaches packed in syrup; strained before weighing
Int 1	2	Spicy beans
Int 1	2	Fresh Honeydew, cantaloupe, watermelon seasoned with tajin spice
Int 1	2	Fresh grapes
Int 1	3	Curry roasted cauliflower
Int 1	3	Fresh grapes
Int 1	3	Seasoned green beans; strained before weighing
Int 1	3	Side salad of green lettuce dressed with Italian vinaigrette; strained before weighing
Int 1	3	Strawberry medley
Int 1	4	Fresh baby carrots
Int 1	4	Uncooked Broccoli and tomato pieces lightly dressed in vinaigrette dressing; strained before weighing
Int 1	4	Strawberries packed in syrup, previously frozen and thawed for serving; strained before weighing
Int 1	4	Whole fresh apple
Int 1	4	Fresh grapes
Int 1	4	Seasoned green beans; strained before weighing
Int 1	4	Japanese vegetable mix including edamame, corn, carrots
Int 1	4	Strawberry medley
Int 1	5	Fresh baby carrots
Int 1	5	Peaches packed in light syrup; strained before weighing
Int 1	5	Canned corn that was steamed; strained before weighing
Int 1	5	Fresh cucumber cut into spears
Int 1	5	Frozen fruit cup treat - Frozen fruit juice from concentrate
Int 1	5	Mashed potatoes served with and without brown or white gravy
Int 2	1	Fresh sliced apples
Int 2	1	Canned pears packed in light syrup; strained before weighing
Int 2	1	Oven baked fries
Int 2	1	Carrots tossed with water and sugar before roasting
Int 2	1	Canned mandarin oranges packed in light syrup; strained before weighing
Int 2	2	Canned corn that was steamed; strained before weighing
Int 2	2	Canned mandarin oranges packed in light syrup; strained before weighing
Int 2	2	Canned pineapple packed in juice; strained before weighing
Int 2	2	Steamed green beans
Int 2	3	Canned peaches packed in light syrup; strained before weighing
Int 2	3	Frozen steamed peas and carrots; strained before weighing
Int 2	3	Served with or without tomatoes; dressing served on side
Int 2	4	Canned peaches packed in light syrup; strained before weighing
Int 2	4	Charro beans
Int 2	4	Whole fresh pear
Int 2	4	Canned pineapple chunks packed in juice; strained before weighing
Int 2	4	Steamed broccoli
Int 2	5	Fresh celery sticks
Int 2	5	Whole apple
Int 2	5	Mixed fruit cocktail
Int 2	5	Sweet potato roasted with butter and sugar
Int 2	5	Whole fresh pear
Control	1	Fresh baby carrots
Control	1	Oven baked fries
Control	1	Canned mandarin oranges packed in light syrup; strained before weighing
Control	2	Fresh grapes
Control	2	Fresh jicama tossed with lime juice served with option packet of tajin seasoning
Control	2	Vegetable blend
Control	3	Sweet potato roasted with butter and sugar
Control	3	Canned pineapple packed in juice; strained before weighing
Control	3	Spinach side salad
Control	4	Fresh banana; peel not included in weighing
Control	4	Refried beans
Control	4	Steamed broccoli
Control	5	Fresh honeydew, cantaloupe, grapes, and pineapple
Control	5	Carrots tossed with water and sugar before roasting
Control	5	Kale side salad
Control	5	Mandarin oranges in light syrup; strained before weighing
Control	6	Mixed fruit cocktail
Control	6	Carrots tossed with water and sugar before roasting
Control	6	Steamed cauliflower

Notes: Int: Intervention School. In the control school, six days of data collection were conducted due to an unscheduled citywide holiday which did not affect the two intervention schools. We chose to measure the 6th day, only under specific circumstances if the school was closed on one day in the week of measurement, or if a >25% of the participating students were absent due to illness (or other reasons) in any of the schools.
